# Peer review of "Role of SIRT-3, p-mTOR and HIF-1α in Hepatocellular Carcinoma Patients Affected by Metabolic Dysfunctions and in Chronic Treatment with Metformin"

_ijms, 2019, doi:10.3390/ijms20061503_

Round 1

Reviewer 1 Report

Summary

The authors tried to elucidate the roles of SIRT-3, p-mTOR and HIF-1α in HCC patients with metabolic dysfunctions from both functional and prognostic perspectives. Findings are quite interesting, such as the differential expressions of SIRT-3 and p-mTOR in HCC with different metabolic conditions/ etiologies/ treatments and the prognostic values of SIRT-3, p-mTOR and HIF-1α in different stages of HCC.

Major points

1.       Patient characteristics are very briefly described in 2.1. More detailed clinical parameters should be summarised in a table, including gender, ages, etiologies (viral: HBV/HCV; metabolic; alcoholic; others), diabetic/ non-diabetic, tumour sizes, single or multiple tumour nodules, metastasis, capsule infiltration/invasion, cirrhosis, portal thrombosis, BCLC staging, TNM staging, and AFP levels, which can be helpful to the readers. The authors can also try to assess the associations of SIRT-3, p-mTOR and HIF-1α with these clinical parameters.

2.       The authors used IHC to evaluate the expression of SIRT-3, p-mTOR and HIF-1α in HCC patients. It is good to see that the expression levels were separately assessed in two different settings (early and advanced). I wonder if there are any differences in expression levels within the positive cohort in the same settings, i.e. strongly positive and weakly positive. If yes, the authors can consider further dividing the positive cases into +, ++, +++, etc.

3.       The authors reported that SIRT-3 and p-mTOR are related to metabolic etiology, presence of T2DM, and treatment with metformin in HCC patients. Despite existing studies showing the putative role of these markers in HCC metabolism, the authors should include non-cancerous/healthy controls of comparable cohort size (diabetic vs non-diabetic; metformin-treated vs insulin-treated) to exclude the possibility that the phenomenon is general in metabolic dysfunctions and not specific to HCC patients with metabolic dysfunctions.

4.       Texts from Line 118 – 127 described the effects of metformin in combination with sorafenib on cell proliferation and apoptosis in three HCC cell lines. What are the objectives of these experiments? How are they related to the main study? Are they to show sorafenib resistance? I’d like very much to have the authors’ rationale behind the study design. Besides, I do not think these texts suit well under the subtitle ‘In vitro effect of metformin on SIRT-3 and p-mTOR expression levels’. The same for Fig 3ABCD to be under the same figure title. The authors may consider modifying the subtitle and figure titles.

5.       WB data (Fig 3E; Line 128- 136) to show the in vitro effect of metformin on expression of the markers are somehow inconclusive but that is the data I understand and as the author have explained in the Discussion.

6.       It is exciting to see the predictive values of SIRT-3 and HIF-1α in early-stage HCC patients and p-mTOR in advanced HCC patients. In Line 158 – 159, there is indeed a significant correlation between SIRT-3 and HIF-1α (Spearman coefficient of 0.42; P = .048). However, if the authors would like to show a ‘direct’ association, additional biological experiments will be required I assume.

Minor points

1.       Axis labels in Fig 2 are too small to see properly.

2.       Texts from Line 97 – 104 describe that there are patients taking insulins whereas in Fig 2 it says no therapy. Please be consistent.

3.       Line 118 – 127: the three HCC cell lines were treated with metformin (0 - 20 mmol/mL) for 48 h. Why and how did the authors choose the metformin concentration range and treatment time? If the authors did some optimization experiments prior to the treatments, please show the data as supplementary.

4.       Line 124 – 127: the authors can show the representative flow cytometry figures in Annexin V/PI assays together with the bar chart (Fig 3D).

5.       The authors mentioned that they used Image J to analyse the Western Blot images (Fig 3E) in Line 320. The quantification can be shown as bar charts with the WB images. If there were replicates, statistical significance should be included.

6.       All the full and unedited WB images should be supplied as supplementary and made available to the public.

7.       I wonder why the authors used vinculin as WB loading controls for SIRT-3 and p-AMPK in Fig 3E as they are not that high in molecular weights.

8.       Line 297: how many cells did you use?

9.       Line 306: the amounts of proteins loaded each should be reported clearly and separately instead of using a range.

Author Response

Major points

Point 1. Patient characteristics are very briefly described in 2.1. More detailed clinical parameters should be summarised in a table, including gender, ages, etiologies (viral: HBV/HCV; metabolic; alcoholic; others), diabetic/ non-diabetic, tumour sizes, single or multiple tumour nodules, metastasis, capsule infiltration/invasion, cirrhosis, portal thrombosis, BCLC staging, TNM staging, and AFP levels, which can be helpful to the readers. The authors can also try to assess the associations of SIRT-3, p-mTOR and HIF-1α with these clinical parameters.

Response 1

As requested by the reviewer, I summarized the clinical informations in a Table (Table 1). This is a retrospective study and unfortunately, some clinical parameters that the reviewer indicated, have not been recoverable. Moreover, in the paragraph 2.2 we described the correlation between the expression levels of the markers and clinical parameters.

Point 2. The authors used IHC to evaluate the expression of SIRT-3, p-mTOR and HIF-1α in HCC patients. It is good to see that the expression levels were separately assessed in two different settings (early and advanced). I wonder if there are any differences in expression levels within the positive cohort in the same settings, i.e. strongly positive and weakly positive. If yes, the authors  can consider further dividing the positive cases into +, ++, +++, etc.

Response 2

As requested by the reviewer, I added a Supplementary Table 1 in which we reported the staining intensity related to the expression of SIRT-3 and p-mTOR in the two setting of HCC patients. We don't describe the intensity of HIF-1α because a strong nuclear expression in all positive cases and no citoplasmic  expression was observed (we added a sentence in line 98-101).

Point 3. The authors reported that SIRT-3 and p-mTOR are related to metabolic etiology, presence of T2DM, and treatment with metformin in HCC patients. Despite existing studies showing the putative role of these markers in HCC metabolism, the authors should include non-cancerous/healthy controls of comparable cohort size (diabetic vs non-diabetic; metformin-treated vs insulin-treated) to exclude the possibility that the phenomenon is general in metabolic dysfunctions and not specific to HCC patients with metabolic dysfunctions.

Response 3

I thank the reviewer for this remark. Unfortunately, I don’t have healthy tissues derived from diabetic subjects or with metabolic syndrome. Thoughout, I recovered the tissue slides of biopsy derived from HCC patients with diabetes or metabolic syndromes, focusing on the differences between tumor and peritumoral tissue. I noted that the expression of the markers resulted negative or slightly positive in the peritumoral tissue.

Point 4. Texts from Line 118 – 127 described the effects of metformin in combination with sorafenib on cell proliferation and apoptosis in three HCC cell lines. What are the objectives of these experiments? How are they related to the main study? Are they to show sorafenib resistance? I’d like very much to have the authors’ rationale behind the study design. Besides, I do not think these texts suit well under the subtitle ‘In vitro effect of metformin on SIRT-3 and p-mTOR expression levels’. The same for Fig 3ABCD to be under the same figure title. The authors may consider modifying the subtitle and figure titles.

Response 4

 I thank the reviewer for this remark. Indeed the subtitle resulted unclear. So I modified the subtitle and the title of Figure 3 to better explain the main object of the experiments performed on HCC cell lines. Regarding the rationale of this study, it derives from our work published previously (Casadei Gardini et al. ) as mentioned in the Introduction and Discussion, where we showed a lower response to sorafenib in HCC patients undergoing chronic therapy with metformin for T2DM in respect with non-diabetic patients or those taking insulin, suggesting an involvement of SIRT-3 in the mechanism of resistance to sorafenib in this setting of patients. To better understand the implication of SIRT-3 and its downstream effectors in the drug resistance mechanism, we investigated the effect of metformin and sorafenib in three HCC cell models.

Point 5. WB data (Fig 3E; Line 128- 136) to show the in vitro effect of metformin on expression of the markers are somehow inconclusive but that is the data I understand and as the author have explained in the Discussion.

Response 5

We  showed a modulation in the expression of the markers, suggesting different mechanisms involved p-mTOR regulation by p-AMPK/SIRT-3 in the three HCC cell lines. We better explained in the Discussion (line 243-248).

Point 6. It is exciting to see the predictive values of SIRT-3 and HIF-1α in early-stage HCC patients and p-mTOR in advanced HCC patients. In Line 158 – 159, there is indeed a significant correlation between SIRT-3 and HIF-1α (Spearman coefficient of 0.42; P = .048). However, if the authors would like to show a ‘direct’ association, additional biological experiments will be required I assume.

Response 6

We agree with the reviewer's remark. The data relative to the direct association between SIRT-3 and HIF-1a, results weak if it is not supported by additional experiments that to date we don't perform  because the study would require further time to plan the precise design and validation phase. For this reason,  we decided to remove the sentence in which we reported the correlation (line 174-177).

Minor points

Point 1. Axis labels in Fig 2 are too small to see properly.

Response 1

The size of Figure 2 has been modified.

Point 2. Texts from Line 97 – 104 describe that there are patients taking insulins whereas in Fig 2 it says no therapy. Please be consistent.

Response 2

I corrected in the revised Fig. 2 “no therapy” with “Insulin” and “therapy” with “metformin”.

Point 3. Line 118 – 127: the three HCC cell lines were treated with metformin (0 - 20 mmol/mL) for 48 h. Why and how did the authors choose the metformin concentration range and treatment time? If the authors did some optimization experiments prior to the treatments, please show the data as supplementary.

Response 3

We have chosen the metformin concentration range and treatment time based on the literature. Various studies, performed on HCC cell lines, reported the same concentrations and time points (Tsai HH et al. Metformin promotes apoptosis in hepatocellular carcinoma through the CEBPD-induced autophagy pathway. Oncotarget. (2017); Zhang HH et al. Metformin in combination with curcumin inhibits the growth, metastasis, and angiogenesis of hepatocellular carcinoma in vitro and in vivo. Mol Carcinog. (2018); Rastegar M et al. Investigating Effect of Rapamycin and Metformin on Angiogenesis in Hepatocellular Carcinoma Cell Line. Adv Pharm Bull. (2018)).

Point 4. Line 124 – 127: the authors can show the representative flow cytometry figures in Annexin V/PI assays together with the bar chart (Fig 3D).

Response 4

I added the representative dot plots for Annexin V/PI in the revised Fig 3 and modified the relative Figure Legend.

Point 5. The authors mentioned that they used Image J to analyse the Western Blot images (Fig 3E) in Line 320. The quantification can be shown as bar charts with the WB images. If there were replicates, statistical significance should be included.

Response 5

I added directly the quantified values above each bands as reported in the revised Fig.3E.

Point 6. All the full and unedited WB images should be supplied as supplementary and made available to the public.

Response 6

I added as Supplementary data the full and unedited WB images.

Point 7. I wonder why the authors used vinculin as WB loading controls for SIRT-3 and p-AMPK in Fig 3E as they    are not that high in molecular weights.

Response 7

We used Vinculin as loading control mostly because it has a different molecular weight  from the other proteins of interest in order to better distinguish between specific bands without interference with non-specific ones. 

Point 8. Line 297: how many cells did you use?

Response 8

I added the number of cells (line 338).

Point 9. Line 306: the amounts of proteins loaded each should be reported clearly and separately instead of using a range.

Response 9

I specified separately the amount of protein loaded: 40 µg for p-AMPK and p-mTOR western blots, which required more protein for bands detection, and 20 µg for SIRT-3 western blots (line 346-347).

Reviewer 2 Report

In this manuscript, the authors determined the expression levels of SIRT-3, p-mTOR and HIF-1α in

hepatocellular carcinoma (HCC) patients, and analyzed their correlation with the presence of metabolic dysfunctions, chronic treatment with metformin and clinical outcome. Their observations are interesting. However, data translation and conclusions are not clear, and experimental data are not sufficient to support them. The following points are, at least, considered.

Major:

1. Please prepare a table summarizing patients characteristics

2. The correlation between the expression levels in late-stage patients and the response rate to sorafenib should be analyzed. 

3. Are there significant differences in the marker expressions (especially SIRT-3) between early and late stages? If the authors do not shown the results in Figure 2, their P values should be described.

4. Lines 913-194 "Our study suggested that SIRT-3 activation may relate to metformin-induced activation of AMPK":

5. The authors described in the Discussion that p-mTOR upregulation in HCC patients with T2DM and those treated with metformin might be caused by insulin resistance. Inulin sensitivity or other related clinical parameters of HCC patients with T2DM should be reports. Did you check autophagy status in HCC patients, for example, by LC-3 or p62?

6. To discuss insulin resistance or metabolic syndrome, it is necessary to investigate the expression in non-tumor tissues. Cancer cells are metabolically reprogrammed.

7. HIF1a is a well-known oncogenic factor. It should be discussed or investigated why higher HIF1a expression resulted in better prognosis.

8. Although all three cell lines were damaged by the combination of sorafenib and metformin, a concomitant change in protein expression after drug treatment was not observed. This suggests that these proteins are not involved in the cytotoxic effects of the drugs. The authors discuss on this issue, and rationalize why these data are necessary to be shown.

Minor:

1. Lines 66-67: "positively regulates SIRT-3 or indirectly by AMPK-independent mechanism" SIRT-3 or what? Please revise the sentence. 

2. Line 114: "ex vivo" should be "in vitro".

3. Font size of Figure 2 is too small to read without magnification, and should be bigger.

4. There are several grammatical errors throughout manuscript. Please have it proof-read again by a professional.

Author Response

Response to Reviewer 2 Comments

Major revisions

Point 1. Please prepare a table summarizing patients characteristics.

Response 1

As requested by the reviewer, I summarized the clinical information of patients in a Table  

(Table 1).

Point 2. The correlation between the expression levels in late-stage patients and the response rate to  sorafenib should be analyzed.

Response 2

I added a Table (Table 3) in which we reported the correlation between the expression levels of the markers in late-stage patients and the response rate to sorafenib with a reference in the text (line 185-187).

Point 3. Are there significant differences in the marker expressions (especially SIRT-3) between early and late stages? If the authors do not shown the results in Figure 2, their P values should be

described.

Response 3

I reported a sentence in the text (line 104-106) relative to the difference in the expression of the markers between the two cohorts of patients.

Point 4. Lines 193-194 "Our study suggested that SIRT-3 activation may relate to metformin-induced activation of AMPK":

 Response 4.

I suppose that the reviewer would request the revision of the sentence. I better explained the

sentence (line 67-68). 

Point 5. The authors described in the Discussion that p-mTOR upregulation in HCC patients with T2DM and those treated with metformin might be caused by insulin resistance. Insulin sensitivity or other related clinical parameters of HCC patients with T2DM should be reports. Did you check autophagy status in HCC patients, for example, by LC-3 or p62?

Response 5

I thank the reviewer for this remark. Indeed the detailed mechanisms linking autophagy and metabolic dysfunction in HCC remain an open question. Recently, it has been demonstrated that metformin induced autophagy through CEBPD upregulation and LC3B and ATG activation, and the combined treatment of metformin and rapamycin enhanced autophagic cell death of HCC (Tsa et al. Oncotarget 2017). It could be very interesting to increase our understanding on the relationship between metabolic dysfunctions and autophagy in HCC patients. Unfortunately, for this work we have not checked the autophagy status in HCC patients, but this suggestion is very appreciated and it will be considered for the future investigations. We added a short sentence in the text (238-240).

Point 6. To discuss insulin resistance or metabolic syndrome, it is necessary to investigate the expression in non-tumor tissues. Cancer cells are metabolically reprogrammed.

Response 6

I thank the reviewer for this remark. Unfortunately, I don’t have healthy tissues derived from diabetic subjects or with metabolic syndrome. Thoughout, I recovered  tissue slides of biopsy derived from HCC patients with diabetes or metabolic syndromes, focusing on the differences between tumor and peritumoral tissue. I noted that the expression of the markers resulted negative or slightly positive in the peritumoral tissue.

Point 7. HIF1a is a well-known oncogenic factor. It should be discussed or investigated why higher HIF1a expression resulted in better prognosis.

 Response 7

I agree with the reviewer's remark. Our observation is in contrast with the literature that we cited in the text and in contrast with the oncogenic role of HIF-1a described to date.

We better discussed in the text as requested by the reviewer (line 268-272).

Point 8. Although all three cell lines were damaged by the combination of sorafenib and metformin, a concomitant change in protein expression after drug treatment was not observed. This suggests that these proteins are not involved in the cytotoxic effects of the drugs. The authors discuss on this issue, and rationalize why these data are necessary to be shown.

Response 8

As reported in the quantification of the bands relative to the proteins investigated (the revised Fig.3), we showed a treatment-induced modulation of their expression. We suggested different mechanisms involved p-mTOR regulation by p-AMPK/SIRT-3 between the three cell lines. We better discussed the western blot data in the discussion (line  244-249).

Minor revisions

Point 1. Lines 66-67: "positively regulates SIRT-3 or indirectly by AMPK-independent mechanism"

SIRT-3 or what? Please revise the sentence. 

Response 1

I revised the sentence.

Point 2. Line 114: "ex vivo" should be "in vitro".

Response 2

 "Ex vivo" is correct because it refers to ex vivo study previously performed. Through I better

 explained the sentence (line 129).

Point 3. Font size of Figure 2 is too small to read without magnification, and should be bigger.

Response 3

I modified the size of the Figure 2.

Point 4. There are several grammatical errors throughout manuscript. Please have it proof-read again by a professional.

Response 4

The manuscript has been re-edited.

Round 2

Reviewer 1 Report

The responses from the authors have well addressed my previous. Based on the revised manuscript and recently supplemented data, I’d like the authors to take a look at the followings before a further decision of acceptance.

Majors:

1.       IHC staining intensity: I’d like to thank the authors for summarising the IHC staining intensity. As we can see, among the positive cohorts, especially SIRT-3-positive patients, the positive intensities varied significantly. Due to the tumour heterogeneity, simply dividing all the cases into just negative and positive can be quite arbitrary and non-representative. I’d encourage the authors to develop a scoring system based on both positive percentage and intensity and re-analyse the statistical significance accordingly.

2.       I understand that the authors don’t own liver tissues derived from non-HCC patients with DM or other metabolic syndromes. What I got from the rebuttal is that the authors retrieved the non-cancerous adjacent liver tissues from HCC patients with DM or metabolic syndromes and noticed that the expression of the said markers remained either negative or slightly positive. The authors should report how many cases of non-cancerous adjacent liver tissues they have examined and provide the statistical analysis accordingly in the manuscript.

3.       The authors’ previous work (PMID: 26513009) reported a lower response to sorafenib in metformin-treated HCC patients combined with T2DM as compared to HCC patients taking insulin or non-diabetic HCC patients. The authors have also mentioned this published work in the Introduction and Discussion. In Figure 3A-C, what we can observe is that combined exposure to metformin and sorafenib induced a greater arrest of cell viability as compared to single drug treatment in the three HCC cell lines. That is to say, metformin-administered HCC cells did not demonstrate a lower response to sorafenib than the non-metformin-treated HCC cells, which is not consistent with the authors’ previous published findings. I understand that there always discrepancies in clinical and in vitro findings but the authors can try to better discuss.

Minors:

1.       Figure 3D: It’s good to see the flow dot plots are now supplied. The authors should relabel the axis names with Annexin V and PI instead of using the colour channel names. Also, percentage should be provided in each quadrant.

2.       Mathematical notation for decimal separator is inconsistent. The authors used ‘.’ and ‘,’ interchangeably. In recently edited Figure 3 and some parts of the text, ‘,’ was used as decimal separator whereas ‘.’ was widely used in texts coming from the previous version. Please be consistent.

Author Response

Response to Reviewer 1 Comments

Majors:

Point 1. IHC staining intensity: I’d like to thank the authors for summarising the IHC staining intensity. As we can see, among the positive cohorts, especially SIRT-3-positive patients, the positive intensities varied significantly. Due to the tumour heterogeneity, simply dividing all the cases into just negative and positive can be quite arbitrary and non-representative. I’d encourage the authors to develop a scoring system based on both positive percentage and intensity and re-analyse the statistical significance accordingly.

Response 1

We thank the reviewer for this remark. We re-analysed the data considering the positive intensity variations reported in Supplementary Table 1 that we modified because there were some mistakes. We considered two statistical methods: the first described by Fred R. Hirsch et al. (The first Epidermal growth factor receptor in non-small-cell lung carcinomas: Correlation between gene copy number and protein expression and impact on prognosis. J Clin Oncol 2003), where the authors assessed by immunohistochemistry the protein expression on a scale from 0 to 400 (percentage of positive cells x staining intensity).

The second reported by Redha Al-Bahrani  et al.  (“Differential SIRT1 expression in hepatocellular carcinomas and cholangiocarcinoma of the liver” Ann Clin Lab Sci. 2015), where the authors used both semi-quantitative methods namely multiplying the percentage of positive tumor cells by the intensity rating according to a previous method validated for non-parametric evaluations. Samples were scored by extent (0/none, 1/1–25%, 2/26–50%, 3/51–75%, 4/76–100% of the tumor cells being positive) and intensity of staining (0/negative, 1/weak, 2/moderate, 3/intense).

The statistical revisions confirmed our data already described in Table 2, 3 and 4. We added a sentence in line 183-185 and in line 199-200, highlighting this aspect and reporting the references.

In the file uploaded, we reported table examples in which we show how the three statistical analyses are comparable.

Moreover, we modified some sentences because they were not precise from a statistical point of view (line 175-177 in the Results; 274-276 in the Discussion and line 44-45 in the Abstract). We also added a sentence in line 325-326 relative to staining intensity (methods -> Immunohistochemistry paragraph).

Point 2. I understand that the authors don’t own liver tissues derived from non-HCC patients with DM or other metabolic syndromes. What I got from the rebuttal is that the authors retrieved the non-cancerous adjacent liver tissues from HCC patients with DM or metabolic syndromes and noticed that the expression of the said markers remained either negative or slightly positive. The authors should report how many cases of non-cancerous adjacent liver tissues they have examined and provide the statistical analysis accordingly in the manuscript.

Response 2

As requested by the reviewer, in HCC cases where non-cancerous adjacent liver tissues was present, we analysed the expression of SIRT-3 and p-mTOR. We added a Supplementary Table 2. Moreover, we described the data reported in Supplementary Table 2 in the Results (131-136) and discussed the data in the Discussion (line 246-251).

Point 3. The authors’ previous work (PMID: 26513009) reported a lower response to sorafenib in metformin-treated HCC patients combined with T2DM as compared to HCC patients taking insulin or non-diabetic HCC patients. The authors have also mentioned this published work in the Introduction and Discussion. In Figure 3A-C, what we can observe is that combined exposure to metformin and sorafenib induced a greater arrest of cell viability as compared to single drug treatment in the three HCC cell lines. That is to say, metformin-administered HCC cells did not demonstrate a lower response to sorafenib than the non-metformin-treated HCC cells, which is not consistent with the authors’ previous published findings. I understand that there always discrepancies in clinical and in vitro findings but the authors can try to better discuss.

Response 3

As highlighted by the reviewer, in our previous clinical works, we reported a lower response to sorafenib in metformin-treated HCC patients combined with T2DM as compared to HCC patients taking insulin or non-diabetic HCC patients. These findings are discussed in a recent work (Lena Schulte et al. Treatment with metformin is associated with a prolonged survival in patients with hepatocellular carcinoma. Liver International 2019). In the pre-clinical studies we showed opposite results when we tested metformin in combination with sorafenib. In the Discussion, we better discussed this discrepancy between our pre- and clinical studies (line 219-223). We believe in the goodness of both our clinical data and those conducted in vitro on cell lines.  Indeed we think that the differences can be justified by the difficulty of reproducing the metabolic condition of patients in vitro.

Minors: 

Point 1. Figure 3D: It’s good to see the flow dot plots are now supplied. The authors should relabel the axis names with Annexin V and PI instead of using the colour channel names. Also, percentage should be provided in each quadrant.

Response 1

As requested by reviewer, the Fig 3D has been modified.

Point 2. Mathematical notation for decimal separator is inconsistent. The authors used ‘.’ and ‘,’ interchangeably. In recently edited Figure 3 and some parts of the text, ‘,’ was used as decimal separator whereas ‘.’ was widely used in texts coming from the previous version. Please be consistent.

Response 2

As highlighted by the reviewer, we modified mathematical notation for decimal separator in the Figure 3, using  ‘.’ as decimal separator.

Reviewer 2 Report

The authors have adequately addressed my comments.

Author Response

Response to Reviewer 2 Comments

Comments and Suggestions for Authors

Point 1. The authors have adequately addressed my comments.

Response 1Throughout we adequately addressed the reviewer's comments, we improved english editing and the results and conclusions thank the reviewers' remarks.

Round 3

Reviewer 1 Report

The authors have well addressed my concerns during the two revisions.