# Supplementary material for: Role of SIRT-3, p-mTOR and HIF-1α in Hepatocellular Carcinoma Patients Affected by Metabolic Dysfunctions and in Chronic Treatment with Metformin
Source: Int J Mol Sci. 2019 Mar 26;20(6):1503. doi: 10.3390/ijms20061503 (PMC6470641; doi:10.3390/ijms20061503)
Supplement: Supplementary file 1 [file ijms-20-01503-s001.pdf]

Table S1. Staining intensity of the SIRT-3 and p-mTOR expression in early-and advantage stage HCC patients.

	**Early-stage HCC patients**		**Advanced-stage HCC patients**	
**Intensity**	**SIRT-3** *No. patient* **(%)**	**p-mTOR** *No. patient* **(%)**	**SIRT-3** *No. patient* **(%)**	**p-mTOR** *No. patient* **(%)**
**0 (absent)**	5 (10.9)	23 (85.2)	4 (19.0)	13 (81.2)
**1+ (weak)**	10 (21.7)	3 (11.1)	2 (9.5)	2 (12.5)
**2+ (moderate)**	19 (41.3)	1 (3.7))	6 (28.6)	0
**3+ (strong)**	12 (26.1)	0	9 (42.9)	1 (6.3)

Table S2. Expression of SIRT-3 and p-mTOR in non-cancerous adjacent liver tissue from HCC patients with metabolic dysfunctions.

	**Non-cancerous adjacent liver tissue**					
	**SIRT-3**			**p-mTOR**		
	**No. patients**	**Negative**	**Slight positive**	**No. patients**	**Negative**	**Slight positive**
**Presence of diabetes and metabolic syndrome**	9	7/9	2/9	5	5/5	0
**Only diabetes**	8	4/8	4/8	3	3/3	0
